# Limit to Child Bearing

**Published:** 1875-01

**Authors:** 


LIMIT OF CHILD BEARING.—In the *Medical Times,* Dr. Fordyce Barker says: "I am not aware that this question has ever been judicially settled in our American courts. But, in conclusion, I feel warranted in stating the proposition that the laws of physiology, the experience of mankind, and the decisions of courts of law, justify a medical witness in declaring that a woman over fifty-five years of age is past the period of child bearing."

